# The Effect of Polysaccharide Colloids on the Thermal Stability of Water-in-Oil Emulsions

**DOI:** 10.3390/polym17060809

**Published:** 2025-03-19

**Authors:** Shunfa Zhao, Ran Wang, Ying Xu, Caiyun Wang, Jun Xu, Pengjie Wang, Yonggang Fu, Jiaqi Su, Hanyu Chai, Jian He, Han Chen

**Affiliations:** 1 School of Food and Biological Engineering, Hefei University of Technology, Hefei 230601, China; zhaosf0621@163.com; 2 Department of Nutrition and Health, China Agricultural University, Beijing 100080, China; wangran@cau.edu.cn (R.W.);; 3 Food Laboratory of Zhongyuan, China Agricultural University, Luohe 462000, China; 4 Inner Mongolia Dairy Technology Research Institute Co., Ltd., Hohhot 010100, China; 5 Inner Mongolia Yi Qi Technology Co., Ltd., Hohhot 010100, China; 6 Inner Mongolia National Center of Technology Innovation for Dairy Co., Ltd., Hohhot 010100, China; 7 Inner Mongolia Yili Industrial Group Co., Ltd., Hohhot 010100, China

**Keywords:** curdlan, processability, reduced fat, thermal stability, W/O emulsion

## Abstract

The preference and demand for low-fat diets have increased due to their health benefits. This study aimed to develop a thermally stable water-in-oil (W/O) emulsion. The addition of 3.75 wt% of polysaccharide colloids, including curdlan gum (CG), kappa-carrageenan (kC), gellan gum (GEG), guar gum (GUG), high-ester pectin (HEP), and carboxymethyl cellulose (CMC), to the aqueous phase resulted in the formation of a gel structure within it. Furthermore, these polysaccharide colloids reduced the excessive mobility of water droplets under high-temperature conditions. The oil phase consisted of anhydrous butter and a lipophilic nonionic surfactant. The emulsion was subjected to a heat treatment at 95 °C for 30 min, and the emulsions before and after the heat treatment were characterized. The results showed that among the above colloidal emulsions, the 3.75 wt% CG emulsion did not show significant changes in viscosity, stability index, mean particle size, friction coefficient, and encapsulation efficiency before and after heat treatment. The 3.75 wt% CG colloid showed the most significant enhancement in the thermal stability of W/O emulsions. This study proposes a novel fat-replacement strategy for products requiring high-temperature processing, such as processed cheese.

## 1. Introduction

Reducing fat intake can help lower the incidence of excessive fat consumption, such as obesity, hypertension, and cardiovascular diseases. As a result, low-fat foods have become more widespread. [1]. Fat is vital in food products, affecting texture, flavor, and appearance. Removing or reducing fat can lead to a decline in product quality. Hence, incorporating fat substitutes into low-fat foods is necessary to offset the quality deficiencies of lacking fat. The most common fat replacers are hydrocolloids. [2]. Some researchers have attempted to incorporate hydrocolloids as fat replacers in cheese, including carrageenan [3], β-glucan [4], microparticulated whey protein [5], and soybean isolate protein [6]. The low-fat cheese products mentioned above showed high moisture content with reduced hardness and springiness.

The particle size of W/O emulsions can be controlled by altering the preparation conditions, which affects the product’s texture, particularly its hardness [2,7]. This emulsion with controlled particle size improves the textural properties of low-fat food products. However, W/O emulsions exhibit poor thermal stability. The movement of water droplets intensified after heating, resulting in destabilization phenomena such as agglomeration, precipitation, and Ostwald ripening, which limit their application in food products [8]. Adding polysaccharide colloids to the aqueous phase increases the viscosity of the aqueous phase and reduces the collision of water droplets, thereby enhancing the stability of the emulsions. Concurrently, it enhances the interactions between the oil-water interface and the polymers in the aqueous phase, strengthening the interface stability and inhibiting destabilization such as agglomeration, aqueous phase precipitation, and fat floating [9,10]. Currently, researchers have begun applying polysaccharides to W/O emulsions. For instance, Iqbal et al. [11] investigated W/O emulsions formulated with high methoxyl pectin, kappa-carrageenan, and starch in the internal aqueous phase, focusing primarily on the gelation process of the polysaccharides and emulsions following heat treatment. Their work revealed that variations in gelation properties among different polysaccharides had distinct effects on emulsion characteristics, offering valuable insights for developing low-fat margarine. Cheng et al. [12] also demonstrated that adjusting the ratio of konjac glucomannan to octenyl succinic anhydride starch could modify the formation and stability of emulsions, providing further insights into utilizing emulsion technology to reduce fat content.

Curdlan gum (CG) is a straight-chain β-1,3-glucan characterized by many intramolecular and intermolecular hydrogen bonds. It is insoluble in most organic solvents, such as water and ethanol, but can be easily dispersed in cold water. It is readily soluble in aqueous solutions that destroy hydrogen bonds, such as sodium hydroxide, trisodium phosphate, tricalcium phosphate, and dimethyl sulfoxide [13]. Kappa-Carrageenan (kC) is a hydrophilic hydrocolloid derived from red algal seaweeds, and the solution can form a thermos-reversible gel, which melts when heated and then reforms upon cooling [14]. Gellan gum (GEG) is a macromolecular linear polysaccharide produced by microbial fermentation, capable of forming a gel that solidifies rapidly, melts at high temperatures, and exhibits good thermal stability [15]. Guar gum (GUG) is a natural polysaccharide with strong water-absorbing capacity, good water solubility, and cross-linking properties, often used as a food thickener [16]. Pectin is a type of soluble dietary fiber, usually used as a gelling and thickening agent; it exhibits a certain degree of stability at high temperatures [17]. Carboxymethyl cellulose (CMC) is a product obtained by chemical modification of natural cellulose, which is easily soluble in water and forms a transparent viscous solution with good adhesion, thickening, and stabilizing effects [18].

This study aims to prepare a W/O emulsion with polysaccharide colloids as the internal aqueous phase, maintaining good stability even under high-temperature treatment. This obtained emulsion can serve as a fat replacer that still effectively substitutes fat in food products requiring high-temperature production.

## 2. Materials and Methods

### 2.1. Materials

Curdlan gum was purchased from Zhejiang Shangfang Biotechnology Co., Ltd. (Jiaxing, China). Kappa-carrageenan and Carboxymethyl cellulose were purchased from Shanghai Maclean’s Biochemical Technology Co., Ltd. (Shanghai, China). Guar gum was purchased from Sigma-Aldrich Trading Co., Ltd. (St. Louis, MO, USA). Gellan gum was purchased from Xinjiang Fufeng Biotechnology Co., Ltd. (Urumqi, China). High-ester pectin was purchased from Shandong Qilu Biotechnology Group Co., Ltd. (Liaocheng, China). Trisodium phosphate was purchased from Shanghai Titan Chem Co., Ltd. (Shanghai, China). Anchor anhydrous butter was purchased from the New Zealand Fonterra Cooperative Group. (Shanghai, China). Polyglycerol polyricinoleate (PGPR-4190) was purchased from Palsgaard Shanghai Food Additives Co., Ltd. (Shanghai, China). Corn oil was purchased from COFCO Fulinmen Co., Ltd. (Weifang, China).

### 2.2. Preparation of Emulsions

W/O emulsions were prepared by emulsifying 40 wt% aqueous phases with 60 wt% oil phases by high-speed shear emulsification. Disperse 1.5 wt%, 2.25 wt%, 3 wt%, 3.75 wt%, and 4.5 wt% of CG in pure water at 35 °C, then add 0.5 mol/L trisodium phosphate solution to adjust the pH of the CG solution to 11.5 and heat to 50 °C in a water bath. 3.75 wt% kC, GEG, HEP, CMC, and GUG were added to pure water and dissolved by magnetic stirring at temperatures of 80 °C, respectively. After dissolution, add a trisodium phosphate solution in the same quantity as the trisodium phosphate solution previously added to the CG solution, and then cool the mixed solution to 50 °C. The same mass of trisodium phosphate was added to pure water, heated to 50 °C, and used to prepare a control emulsion without polysaccharide colloids. The oil phase was added 1.7 wt% PGPR to anhydrous butter at 50 °C, and then the mixture was heated to 95 °C in an oil bath. The emulsions were obtained by using a high-speed shear emulsifier (A25, Shanghai Ouhor Machinery Equipment Co., Ltd., Shanghai, China) to dissolve and disperse the emulsifier by shearing the oil phase for 1 min at 10,000 rpm; the aqueous phase was added to the oil phase and sheared for 5 min. The emulsions were cooled to room temperature, heated in an oil bath at 95 °C for 30 min, and then cooled to room temperature for testing.

### 2.3. General Appearance

For the emulsions before and after heat treatment, 20 mL samples were placed in glass bottles, and pictures of their appearance were taken.

### 2.4. Optical Microscopy and Droplet Size

The emulsions were diluted 10 times with corn oil before and after heat treatment. They were vortexed to ensure an even dispersion of the emulsion particles in the corn oil. Then, 5 μL droplets were placed on slides and photographed using an optical microscope (EX21, Ningbo Sunny Optical Instrument Co., Ltd., Ningbo, China) with a 100 × oil immersion objective. The mean particle size of the emulsions was measured using ImageJ 1.53a software. More than 100 random droplets from each sample were analyzed, and the droplet sizes were recorded and expressed as the mean particle size. The change in mean particle size before and after heat treatment was then compared.

### 2.5. Encapsulation Efficiency (EE)

The EE was modified according to the method described by Pu et al. [19]. Before and after heating, the emulsions were centrifuged at 3000× *g* for 10 min at 40 °C using a high-speed refrigerated centrifuge (H4-20KR, Hunan Kecheng Instrumentation Equipment Co., Ltd., Changsha, China). Then, the centrifuged aqueous precipitate was weighed to determine the mass of the unencapsulated aqueous phase. Subsequently, the mass of the encapsulated aqueous phase was calculated by subtracting the mass of the unencapsulated aqueous phase from the total initial aqueous phase mass. The EE was defined as the ratio of the encapsulated aqueous phase’s mass to the emulsion’s total initial aqueous phase mass, expressed as a percentage. The following formula calculated EE:

(1) $$EE\;(\%)=\frac{A_{0}-A_{1}}{A_{0}}$$

where *A*_1_ refers to the amount of unencapsulated aqueous phase in the oil phase and *A*_0_ represents the amount of aqueous phase added to the W/O emulsions.

### 2.6. Turbiscan Stability Index (TSI)

TSI changes of emulsions before and after heat treatment were detected using a Turbiscan Stability Analyzer (France Formulaction Company, Toulouse, France). Analyzing the phase separation trend of emulsions by detecting the intensity of scattered light and backscattered light of emulsion samples over time [20]. TSI was measured with reference and modification to Li et al. [21]. Take 20 mL emulsion in Turbiscan special cylindrical glass. Scan at 50 °C for 4 h, with a frequency of every 10 min. The TSI values obtained before and after heat treatment were compared. Estimating the overall stability of emulsion before and after heating by TSI. The following formula calculated TSI:

(2) $$TSI=\sqrt{\frac{\sum_{i=1}^{n}{\left(x_{i}-x_{BS}\right)}^{2}}{n-1}}$$

where *x_i_* represents the backscattering for each measurement, *x_BS_* is the average of *x_i_*, and *n* is the scan number.

### 2.7. Viscosity

The rheological properties of the emulsions were evaluated using a mechanical rheometer (MCR302e, ANTON PAAR GMBH, Graze, Austria). TSI was measured with reference and modification to Lu et al. [22]. Each sample was placed in a concentric cylinder geometry with a rotating inner cylinder diameter of 26.7 mm and a stationary outer cylinder diameter of 28.9 mm. The change in emulsion viscosity before and after thermal treatment was compared over a range of shear rates from 0.1 to 100 s^−1^ at 40 °C.

### 2.8. Friction Coefficient

The emulsion’s friction coefficient was measured using a coefficient of friction meter (MXD-02, Jinan Languang Electromechanical Technology Co., Ltd., Jinan, China). Measurement of the coefficient of friction is based on Kapanyk’s method with some modifications [23]. A certain amount of emulsion was applied to the surface of the sliding platform to ensure that the sample was uniformly distributed and that the temperature of the emulsion sample was 37 °C. The other slider surface was brought into contact with the emulsion sample, and the test equipment was activated to allow the two surfaces to slide relative. A force transducer measured the friction between the slider and the sliding platform at a speed of 100 mm/min. The change in friction coefficient before and after heat treatment was compared.

### 2.9. Rheological Measurements

The dynamic rheological properties of the polysaccharide solution were measured using a rotational rheometer (MCR302e, ANTON PAAR GMBH, Graz, Austria with a parallel plate (PP50, 50 mm diameter). The gap between the plate and polysaccharide solutions was fixed at 1 mm.

#### 2.9.1. Frequency Sweep

The dynamic oscillatory frequency sweeps were performed following the methodology of Zhu et al. [24] with modifications. Frequency sweeps were conducted across an angular frequency range of 0.1–100 rad/s at 25 °C under a constant strain of 1% within the linear viscoelastic region. The storage modulus (G′), loss modulus (G″), and phase angle (tan δ) of the polysaccharide solutions were measured under these standardized conditions.

#### 2.9.2. Temperature Scanning

The method was adapted from Liu et al. [25] with modifications. To minimize moisture evaporation, a thin layer of low-viscosity silicone oil was applied to the sample periphery. The polysaccharide solution was heated from 25 °C to 95 °C at a rate of 5 °C/min under oscillatory shear conditions (1 Hz frequency, 1% strain), followed by a 30 min isothermal hold at 95 °C. The G′, G″ and tan δ of the polysaccharide solutions were measured. The G′ of the polysaccharide solutions were compared before and after heating at 95 °C.

### 2.10. Statistical Analysis

All experiments were repeated three times. Data processing and analysis of significance were performed using SPSS 26.0. The experimental results were expressed as mean ± standard deviation, and differences between groups were analyzed using one-way ANOVA, with *p* < 0.05 indicating significant differences.

## 3. Results and Discussion

### 3.1. Effect of CG Concentration on the Thermal Stability of Emulsions

**Figure 1:**
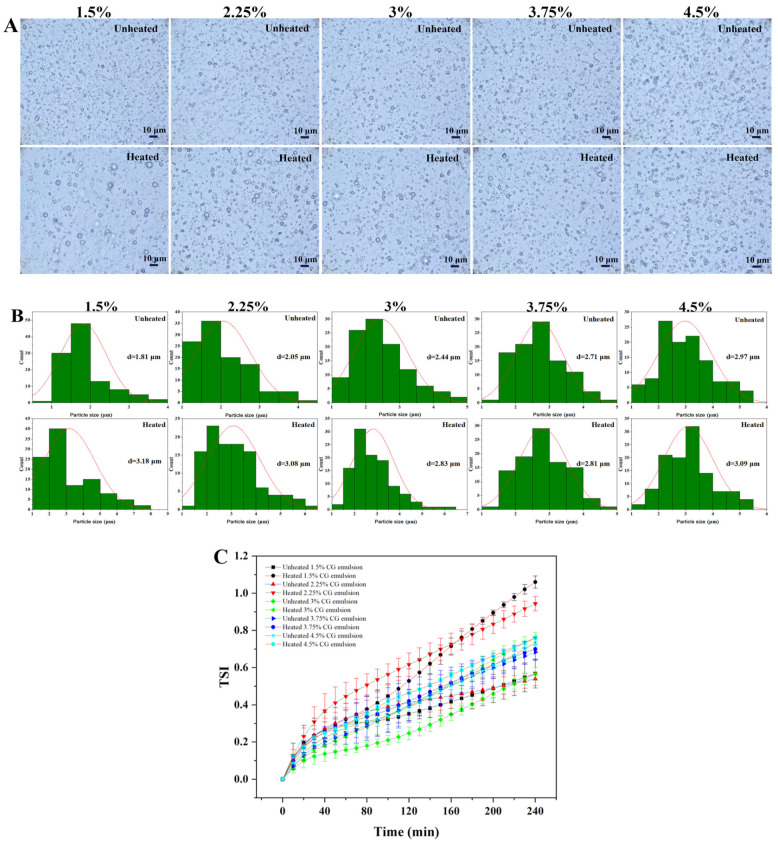
(**A**) Microscopy photographs, (**B**) particle size, the red line represents the normal distribution curve of particle size, and (**C**) TSI of unheated and heated emulsions containing 1.5 wt%, 2.25 wt%, 3 wt%, 3.75 wt%, and 4.5 wt%CG in the aqueous phase.

The particle size of the emulsion is closely related to its stability [26]. The microscopic photographs of the emulsions are shown in Figure 1A. After heat treatment, 1.5 wt%, 2.25 wt%, and 3 wt% CG emulsions showed an increase in particle size. The particle sizes of 3.75 wt% and 4.5 wt% CG emulsions showed minimal variation (Figure 1B). Generally, higher CG concentrations led to smaller particle size increases post-heating, suggesting that CG inhibits agglomeration during heat treatment, thereby maintaining stability.

The changes in TSI before and after heat treatment are shown in Figure 1C. TSI is an index used to assess the overall physical stability of emulsions, with lower values indicating better stability [27]. Before heating, the TSI values of 1.5 wt%, 2.25 wt%, and 3 wt% CG emulsions were lower than those of 3.75 wt% and 4.5 wt% CG emulsions. However, post-heating, the TSI of the lower-concentration CG emulsions (1.5–3 wt%) increased significantly, surpassing the higher-concentration groups. Notably, the 3.75 wt% CG emulsion exhibited the lowest TSI values and the most minor TSI change post-treatment. The 3.75 wt% CG emulsion demonstrated optimal stability. Therefore, this concentration was selected for subsequent thermal stability comparisons with CG, GEG, HEP, CMC, and GUG emulsions.

### 3.2. Appearance Observation of Emulsions

**Figure 2:**
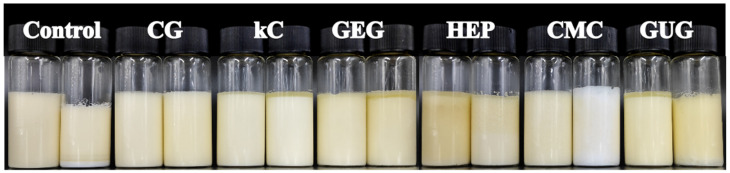
The appearance of unheated and heated emulsions containing polysaccharide-free colloids, 3.75 wt% CG, 3.75 wt% kC, 3.75 wt% GEG, 3.75 wt% HEP, 3.75 wt% CMC, and 3.75 wt% GUG in the aqueous phase. The left side of each group of emulsions was unheated, and the other was heated.

The appearance of emulsions before and after heat treatment is depicted in Figure 2. After heating, all emulsion groups showed varying degrees of separation between the aqueous and oil phases. Specifically, the polysaccharide-free colloidal emulsion boiled over, leading to stratification, which indicated high mobility of the aqueous phase and poor thermal stability. The GUG emulsion exhibited significant colloidal precipitation settling at the bottom, while the GEG, kC, and HEP emulsions showed upward separation of the oil phase, resulting in uneven appearances. The CMC emulsion turned white, indicating substantial precipitation of polysaccharide colloids. In contrast, the CG emulsion maintained its original appearance with no noticeable color change or precipitation, demonstrating superior thermal stability.

### 3.3. Analysis of Optical Microscopy and Particle Size

**Figure 3:**
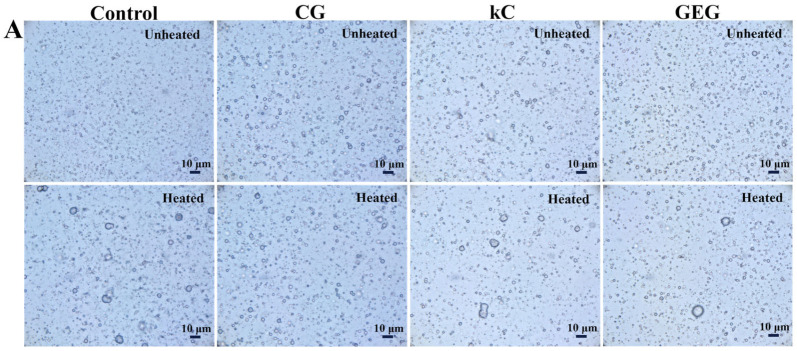

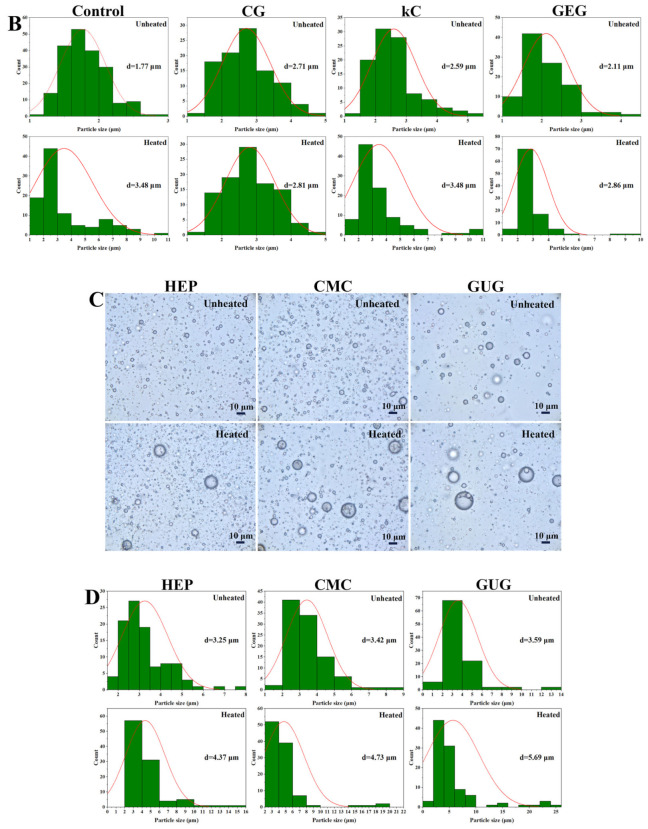
(**A**,**C**) Microscopy photographs, and (**B**,**D**) particle size of unheated and heated emulsions containing polysaccharide-free colloids, 3.75 wt% CG, 3.75 wt% kC, 3.75 wt% GEG, 3.75 wt% HEP, 3.75 wt% CMC, and 3.75 wt% GUG in aqueous phase. Where, the red line represents the normal distribution curve of particle size.

The microscopic photographs of the emulsions are shown in Figure 3A,C. It can be observed that the polysaccharide-free colloidal emulsion initially exhibited the smallest particle size. However, after heat treatment, its particles agglomerated, with a significant increase in size from 1.77 ± 0.32 μm to 3.48 ± 2.06 μm (Figure 3B), indicating poor thermal stability. Similarly, kC, GEG, HEP, CMC, and GUG emulsions showed particle agglomeration and enlarged particles post-heating. In contrast, no significant particle enlargement was observed in CG emulsions (Figure 3A,C). The mean particle size of kC, GEG, HEP, CMC, and GUG emulsions before and after heating were increased from 2.59 ± 0.72 μm, 2.11 ± 0.57 μm, 3.25 ± 1.08 μm, 3.42 ± 1.18 μm and 3.59 ± 1.94 μm to 3.48 ± 1.86 μm, 2.86 ± 1.11 μm, 4.37 ± 2.25 μm, 4.73 ± 2.89 μm, 5.69 ± 4.92 μm, respectively. The CG emulsion displayed the most minor change, increasing only from 2.71 ± 0.67 μm to 2.81 ± 0.69 μm (Figure 3B,D). These results suggest particle motion during heating-induced collisions and oil-phase agglomeration, increasing particle size [11,28]. Notably, the kC emulsion exhibited a rise in non-spherical particles post-heating, likely due to kC colloid aggregation, further confirming its poor thermal stability. Conversely, the CG emulsion showed a mere 3.69% increase in particle size, demonstrating its ability to inhibit agglomeration and enhance thermal stability. In contrast, GUG, HEP, and CMC emulsions displayed substantial particle size growth, indicating weaker thermal resistance.

### 3.4. Analysis of Encapsulation Efficiency

**Figure 4:**
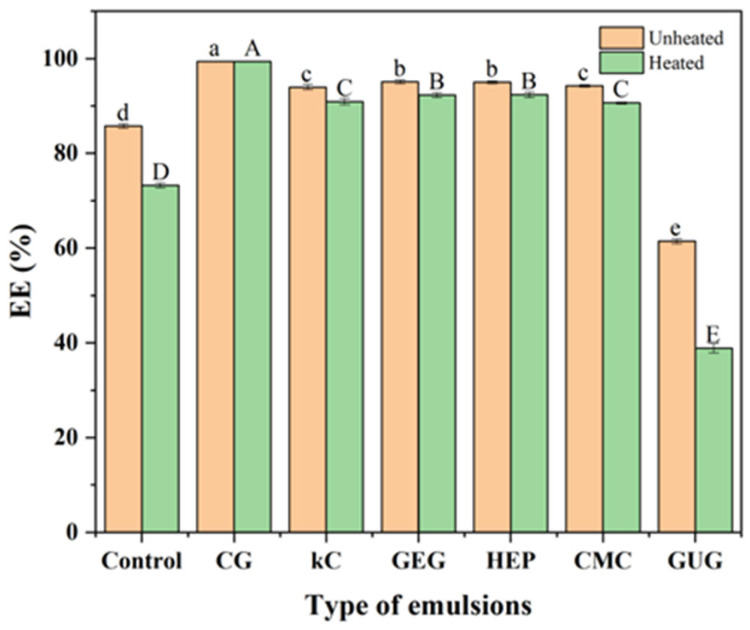
The EE of unheated and heated emulsions containing polysaccharide-free colloids, 3.75 wt% CG, 3.75 wt% kC, 3.75 wt% GEG, 3.75 wt% HEP, 3.75 wt% CMC, and 3.75 wt% GUG in aqueous phase. Where, different superscript lowercase letters indicate significant differences in the EE of unheated emulsions, while uppercase letters indicate significant differences in the EE of heated emulsions.

The EE of the emulsions was measured by centrifugation. During this process, polysaccharide colloids not encapsulated by the oil phase were separated under centrifugal force. The changes in EE before and after heat treatment are shown in Figure 4. The EE of kC, GEG, HEP, and CMC emulsions was similar to each other and higher than that of the polysaccharide-free colloidal emulsion, indicating that polysaccharide colloids enhance EE. Previous studies suggest that biopolymers in the internal aqueous phase improve the EE of W/O/W emulsions, and PGPR may synergize with biopolymers to further enhance EE [29,30].

The low EE of certain emulsions may be attributed to incomplete encapsulation of polysaccharide colloids by the oil phase or their tendency to precipitate from the oil-water interface. The CG emulsion exhibited minimal EE variation before and after heating, maintaining a higher EE than other emulsions. The enhanced stability of the CG emulsion is likely due to its ability to preserve the W/O phase structure during centrifugation. This preserved structure prevents aqueous phase precipitation from the oil–water interface.

### 3.5. Analysis of Turbiscan Stability Index

**Figure 5:**
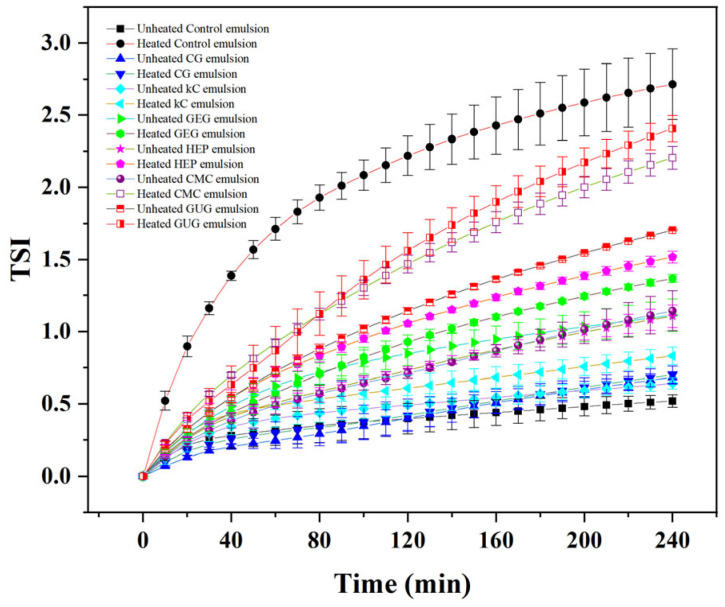
The TSI of unheated and heated emulsions containing polysaccharide-free colloids, 3.75 wt% CG, 3.75 wt% kC, 3.75 wt% GEG, 3.75 wt% HEP, 3.75 wt% CMC, and 3.75 wt% GUG in aqueous phase.

The changes in TSI of emulsions before and after heat treatment are shown in Figure 5. The final TSI of the polysaccharide-free colloidal emulsion increased from 0.52 ± 0.43 to 2.71 ± 0.24 during heating. Although the polysaccharide-free emulsion initially exhibited the lowest TSI before heating, its final TSI reached the highest value post-treatment, indicating poor thermal stability. The significant increase in TSI suggests intense water molecule movement within the emulsion and post-heating agglomeration, as confirmed by optical microscopy (Figure 3A).

Compared to CG, kC, GEG, HEP, CMC, and GUG emulsions, the TSI variation before and after heat treatment in polysaccharide-free emulsions was more pronounced, aligning with its highest final TSI. These results demonstrate that the addition of polysaccharide colloids improves emulsion thermal stability. Jiang et al. [31] reported similar results with emulsions using complexes of soy protein isolate and soy hull polysaccharide, in which they found enhanced viscoelasticity and reduced TSI values of the emulsions along with increased concentration of soy hull polysaccharide. Specifically, the addition of CG reduced the final TSI of the emulsion after heat treatment from 2.71 ± 0.24 to 0.70 ± 0.06, representing the most significant TSI decrease among all tested colloids. The CG emulsion showed only a minor TSI increase from 0.68 ± 0.04 to 0.70 ± 0.06 post-treatment, the slightest change observed (Figure 5). In contrast, kC, GEG, HEP, CMC, and GUG emulsions exhibited higher post-heating TSI values, indicating inferior thermal stability compared to CG.

### 3.6. Analysis of Viscosity

**Figure 6:**
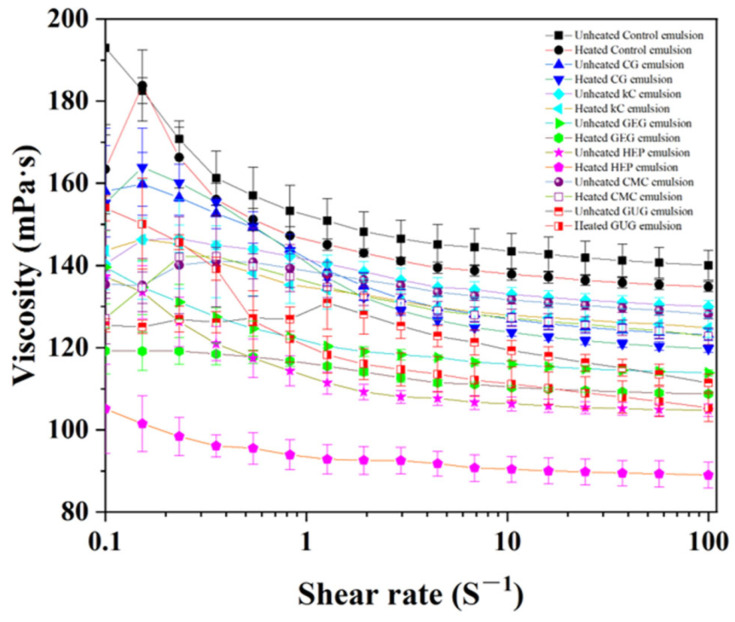
The viscosity of unheated and heated emulsions containing polysaccharide-free colloids, 3.75 wt% CG, 3.75 wt% kC, 3.75 wt% GEG, 3.75 wt% HEP, 3.75 wt% CMC, and 3.75 wt% GUG in aqueous phase.

After heat treatment, the decreased stability of the emulsions induces changes in particle size and aqueous phase precipitation, both of which contribute to altered viscosity in W/O emulsions. An increase in emulsion viscosity reduces the mobility of dispersed-phase molecules and lowers the likelihood of particle collisions, thereby enhancing system stability [32]. The viscosity changes of emulsions before and after heat treatment are shown in Figure 6. The highest viscosity observed in the polysaccharide-free colloid emulsion may be attributed to its smallest particle size [33], as smaller particles exhibit a larger specific surface area, strengthening interparticle interactions and increasing fluid resistance, ultimately elevating viscosity. However, when the particle size of the emulsion increased after heat treatment, the viscosity decreased. Additionally, the results indicate that polysaccharide colloids are a key factor influencing emulsion viscosity.

The viscosity of emulsions prepared from different polysaccharide colloids decreased with increasing shear rate, exhibiting the phenomenon of shear thinning [34]. This property suggests that W/O emulsions were pseudoplastic fluids, resulting in the application of shear and the separation of flocculated oil droplets, leading to a change in the microstructure [35,36]. The CG, kC, and CMC emulsions showed high viscosity. During the stability analysis of emulsions before and after heat treatment at 50 °C for 4 h, it was observed that CG, kC, and CMC emulsions had lower TSI before heat treatment. The lower TSI was attributed to their higher viscosity (Figure 6), which delays the settlement of emulsion particles and reduces the uplift rate of the oil phase, thereby inhibiting emulsion delamination and enhancing its stability [37]. The higher viscosity of kC emulsion was due to the higher affinity of kC for water molecules, which may improve droplet interactions and thus increase the viscosity of the emulsion [38]. Before and after heat treatment, it can be observed from optical microscopy that kC emulsion has more nonspherical colloidal particles (Figure 3A), and these polysaccharide colloids were dispersed in the emulsion, which may also increase the viscosity of the emulsion. After treating CG with trisodium phosphate solution, the molecular structure of CG was disrupted, exposing more hydrogen bonds. Therefore, the hydrophilicity was increased, which may improve droplet interactions [39]. From the general appearance of the emulsion, it can be observed that more CMC colloids are dispersed in the emulsion (Figure 2), which may lead to a CMC emulsion with high viscosity.

Comparison of viscosity changes in emulsions stabilized with different polysaccharide colloids before and after heat treatment revealed that all formulations exhibited varying degrees of viscosity reduction. The CG emulsion demonstrated the most minor viscosity change, decreasing from 123.16 ± 1.37 mPa·s to 119.75 ± 0.90 mPa·s after heat treatment at a shear rate of 100 s^−1^. Whereas, the viscosity of polysaccharide-free colloid, kC, GEG, HEP, CMC and GUG emulsions decreased from 140.02 ± 3.61 mPa·s, 130.03 ± 1.44 mPa·s, 113.89 ± 0.71 mPa·s, 104.78 ± 1.50 mPa·s, 128.10 ± 1.10 mPa·s and 111.40 ± 2.21 mPa·s decreased to 134.80 ± 1.19 mPa·s, 124.84 ± 2.34 mPa·s, 108.77 ± 0.26 mPa·s, 89.01 ± 3.14 mPa·s, 122.80 ± 2.00 mPa·s, and 105.38 ± 3.40 mPa·s. The viscosity decreases were higher than that of the CG emulsion. These reductions exceeded that of the CG emulsion, potentially attributable to increased particle size and polysaccharide colloid sedimentation within the emulsions. The minimal viscosity change in the CG emulsion post-treatment indicates superior thermal stability compared to other formulations.

### 3.7. Analysis of Friction Coefficient

**Figure 7:**
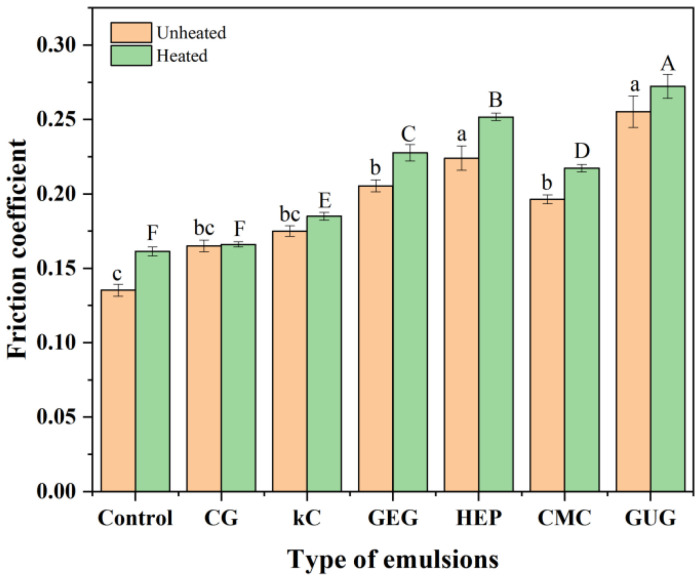
The friction coefficient of unheted and heated emulsions containing polysaccharide-free colloids, 3.75 wt% CG, 3.75 wt% kC, 3.75 wt% GEG, 3.75 wt% HEP, 3.75 wt% CMC, and 3.75 wt% GUG in aqueous phase. Where, different superscript lowercase letters indicate significant differences in the friction coefficient of unheated emulsions, while uppercase letters indicate significant differences in the friction coefficient of heated emulsions.

The friction coefficient depended on particle size, deformation capacity, volume fraction, and viscosity, which could serve as indicators for evaluating the thermal stability of emulsions [40]. Changes in the emulsion’s particle size and the precipitation of polysaccharide colloids resulted in variations in the friction coefficient. Figure 7 shows the changes in friction coefficient before and after heat treatment. It can be observed that the friction coefficient of all groups increased to varying degrees after heat treatment, a phenomenon linked to the increase in particle size and polysaccharide colloid precipitation. The friction coefficients of GEG, HEP, CMC, and GUG emulsions were higher than those of CG and kC emulsions, and their post-treatment increases were also more pronounced. The EE results (Figure 4) indicate that more polysaccharide colloids precipitated in GEG, HEP, CMC, and GUG emulsions. Notably, the highest friction coefficient in the GUG emulsion corresponded to the lowest EE, suggesting a correlation between EE and friction coefficient. Previous studies have demonstrated that incorporating components with slightly higher viscosity into emulsions can reduce the friction coefficient within boundary and mixed lubrication regimes [41,42]. This study showed CG and kC emulsions exhibited higher viscosities, corresponding to lower friction coefficients. The friction coefficient of the CG emulsion was lower than that of the kC emulsion, and its post-treatment increase was also minor. These results demonstrate that polysaccharide colloid precipitation was minimal in the CG emulsion, and the emulsion-maintained stability during heat treatment.

### 3.8. Analysis of Frequency Sweep

Under continuous sinusoidal stress, polysaccharides primarily exhibit solid elasticity and liquid viscosity characteristics, referred to as the storage modulus (G′) and loss modulus (G″), respectively. G′ characterizes the stiffness of the sample, reflecting the strength of interactions between atoms, molecules, and ions, and serves as a measure of the polysaccharide solution’s ability to resist elastic deformation. G″ reflects the sample’s viscosity and positively correlates with water-holding capacity [25]. The phase angle (tan δ = G″/G′, where δ is the loss angle) quantifies the dominance of viscoelastic behavior: tan δ > 1 primarily exhibits viscous behavior, while tan δ < 1 predominantly demonstrates elastic behavior [43].

**Figure 8:**
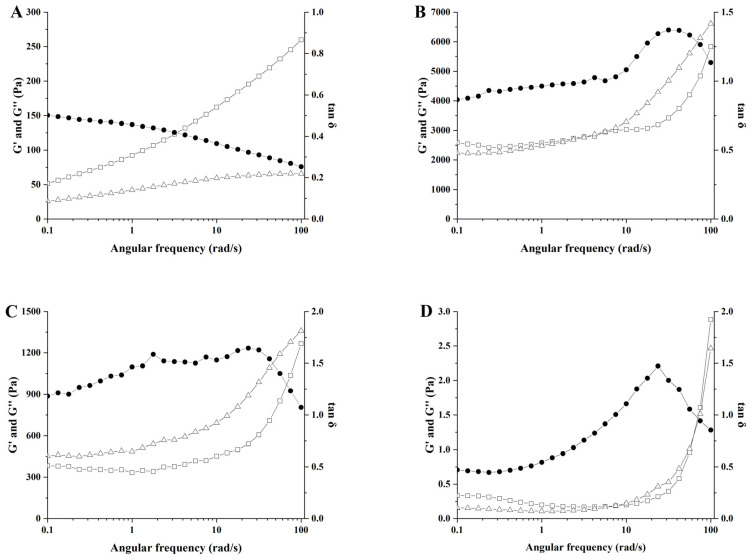

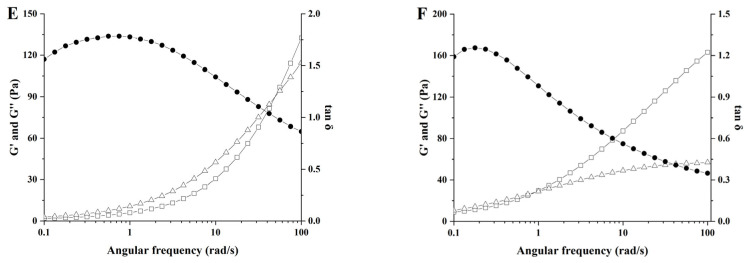
The effect of angular frequency (0.1–100 rad/s) on the storage modulus G′ (□), loss modulus G″ (∆) and phase angle tan δ (●) at 25 °C of different polysaccharide solutions. (**A**) 3.75 wt% CG solution; (**B**) 3.75 wt% kC solution; (**C**) 3.75 wt% GEG solution; (**D**) 3.75 wt% HEP solution; (**E**) 3.75 wt% CMC solution; (**F**) 3.75 wt% GUG solution.

The results of G′, G″ and tan δ for various polysaccharide solutions across an angular frequency range of 0.1–100 rad/s are illustrated in Figure 8A–F. The G′ and G″ values of all polysaccharide solutions increased with rising frequency, indicating that these polysaccharides are viscoelastic materials. CG solution exhibited G′ consistently higher than G″ across the entire frequency range (0.1–100 rad/s), with tan δ < 1, indicating predominantly solid-like elastic behavior. For kC and HEP solutions, G′ exceeded G″ at low angular frequencies, reflecting solid-like elasticity. However, as angular frequency increased, G″ surpassed G′, signifying a transition from solid-like elasticity to liquid-like viscous dominance. This shift arises from disrupting polysaccharide molecular networks in aqueous solutions, transforming the elastic matrix into a fluid-dispersed state. This trend is consistent with findings by Iqbal et al. [11]. Conversely, CMC and GUG solutions displayed inverse characteristics to kC and HEP. At low angular frequencies, G″ predominated over G′, transitioning to G′ > G″ with increasing frequency, thus exhibiting a liquid-to-solid viscoelastic shift. In contrast, the GUG solution-maintained G″ < G′ throughout the 0.1–100 rad/s range, demonstrating persistent fluid-like characteristics. A comparison of the changes in G′ and G″ among different polysaccharide solutions within an angular frequency range of 0.1–100 rad/s revealed that the CG solution exhibited pronounced gel-like characteristics. This behavior enhances the efficacy of the CG solution as the aqueous phase in W/O emulsions by effectively reducing droplet mobility, suppressing the tendency of droplets to aggregate and undergo phase separation, and improving the thermal stability of the emulsion system.

### 3.9. Analysis of Temperature Scanning

**Figure 9:**
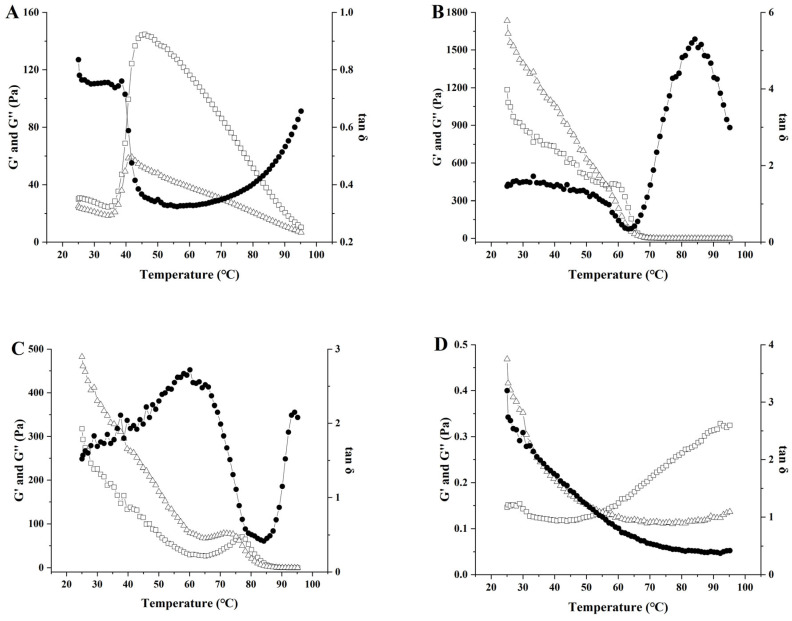

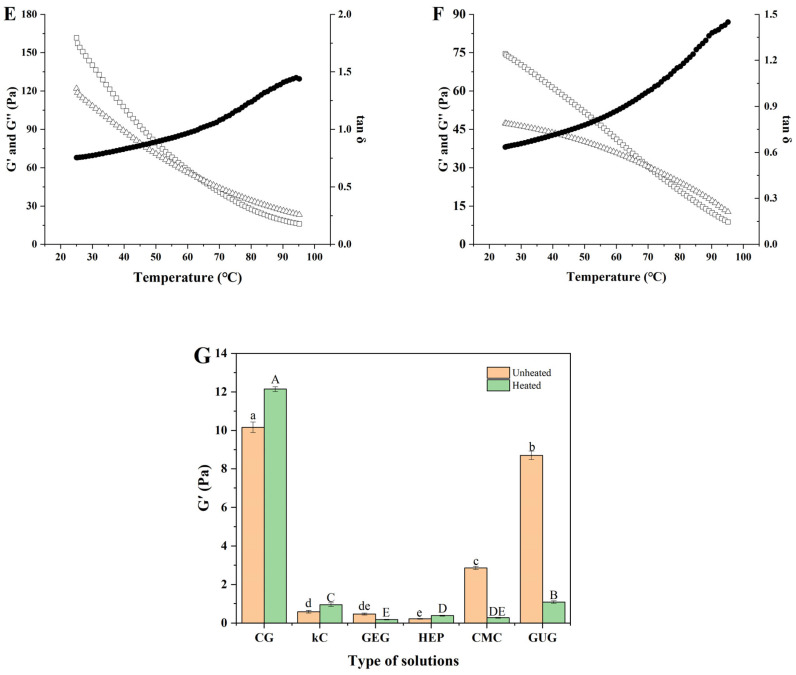
The effect of temperature (25–95 °C) on the storage modulus G′ (□), G″ (∆) and phase angle tan δ (●) of different polysaccharide solutions. (**A**) 3.75 wt% CG solution; (**B**) 3.75 wt% kC solution; (**C**) 3.75 wt% GEG solution; (**D**) 3.75 wt% HEP solution; (**E**) 3.75 wt% CMC solution; (**F**) 3.75 wt% GUG solution; (**G**) Storage modulus G′ before and after heat treatment of polysaccharide solutions, different superscript lowercase letters indicate significant differences in the G′ of unheated solutions, while uppercase letters indicate significant differences in the G′ of heated solutions.

The results of G′, G″ and tan δ for various polysaccharide solutions heated from 25 °C to 95 °C are shown in Figure 9A–F. The kC, GEG, CMC, and GUG solutions all exhibited decreasing trends in both G′ and G″ with increasing temperature, which correlates with structural degradation of polysaccharides under elevated temperatures [44]. At 95 °C, these solutions demonstrated G″ values exceeding G′ with tan δ > 1, displaying liquid-like behavior. In contrast, the HEP solution showed a gradual increase in G′ relative to G″ during heating, though its low G′ value indicated weak gel strength. The CG solution-maintained G′ > G″ throughout the 25–95 °C temperature range with tan δ < 1, exhibiting solid-like characteristics.

Figure 9G presents the G′ values of polysaccharide solutions before and after 30 min of heat treatment at 95 °C. The CG solution demonstrated a relatively high storage modulus at 95 °C, with further G′ enhancement post-treatment. G′ positively correlates with gel strength [45], suggesting improved structural integrity. Other polysaccharide solutions showed significantly lower G′ values than CG before and after heating, predominantly maintaining liquid-like properties indicative of weak gel strength. These rheological characteristics imply that W/O emulsions prepared using kC, GEG, CMC, GUG, or HEP solutions as the internal aqueous phase may exhibit poor stability during 95 °C processing, potentially due to increased fluidity of the aqueous phase under thermal stress.

## 4. Conclusions

The stability changes of W/O emulsions prepared with six polysaccharides (CG, kC, GEG, HEP, CMC, and GUG) as internal aqueous phases were compared before and after heat treatment. Characterizations were conducted, including particle size, TSI, EE, viscosity, and friction coefficient. Experimental results demonstrated distinct differences in thermal stability enhancement among polysaccharides. The 3.75 wt% CG emulsion demonstrated minimal particle size variation (2.71 ± 0.67 μm to 2.81 ± 0.69 μm) and the minor TSI change (0.68 ± 0.04 to 0.70 ± 0.06) following heat treatment, exhibiting optimal enhancement of thermal stability W/O emulsions. Dynamic rheological analysis revealed that 3.75 wt% CG solution-maintained G′ > G″ throughout experimental conditions, exhibiting solid-like elastic behavior, whereas other polysaccharide solutions displayed transitions between elastic-dominated and viscous-dominated characteristics. At 95 °C, the 3.75 wt% CG solution demonstrated superior gel properties with the highest G′ value. This enhanced viscoelasticity restricted internal aqueous phase mobility in CG-stabilized emulsions under thermal stress, effectively inhibiting agglomeration and phase separation. However, the impacts of environmental factors such as pH and ionic strength on emulsion physicochemical properties and stability require further investigation.

## Data Availability

Data are contained within the article.

## Abbreviations

The following abbreviations are used in this manuscript:

CG	curdlan gum
kC	kappa-carrageenan
GEG	gellam gum
HEP	high-ester pectin
CMC	carboxymethyl cellulose
GUG	guar gum
TSI	Turbiscan stability index
EE	encapsulation efficiency
G′	storage modulus
G″	loss modulus
tan δ	phase angle
